# Exploring the Relevance of Green Space and Epidemic Diseases Based on Panel Data in China from 2007 to 2016

**DOI:** 10.3390/ijerph16142551

**Published:** 2019-07-17

**Authors:** Lingbo Liu, Yuni Zhong, Siya Ao, Hao Wu

**Affiliations:** 1 Department of Urban Planning, School of Urban Design, Wuhan University, Wuhan 430072, China; 2 Department of Graphics and Digital Technology, School of Urban Design, Wuhan University, Wuhan 430072, China

**Keywords:** urban green space, public green space, green space coverage, epidemic diseases, panel data model

## Abstract

Urban green space has been proven effective in improving public health in the contemporary background of planetary urbanization. There is a growing body of literature investigating the relationship between non-communicable diseases (NCDs) and green space, whereas seldom has the correlation been explored between green space and epidemics, such as dysentery, tuberculosis, and malaria, which still threaten the worldwide situation of public health. Meanwhile, most studies explored healthy issues with the general green space, public green space, and green space coverage, respectively, among which the different relevance has been rarely explored. This study aimed to examine and compare the relevance between these three kinds of green space and incidences of the three types of epidemic diseases based on the Panel Data Model (PDM) with the time series data of 31 Chinese provinces from 2007 to 2016. The results indicated that there exists different, or even opposite, relevance between various kinds of green space and epidemic diseases, which might be associated with the process of urban sprawl in rapid urbanization in China. This paper provides a reference for re-thinking the indices of green space in building healthier and greener cities.

## 1. Introduction

Humans are facing health challenges due to congested spaces and polluted environments within the contemporary process of planetary urbanization [1]. Urban green space has thus become an effective tool for planning healthy cities, offering not only critical ecosystem services but also significant physical and mental health benefits [2]. 

Diseases threatening human health are classified into epidemics and non-communicable diseases (NCDs). NCDs, such as diabetes and cancers, dominate the global burden of diseases [3]. There is a growing body of literature investigating the relation between green space and NCDs, such as mental illness [4,5,6,7], asthma and allergies [8,9,10], stress restoration [11], focusing on the capability of improving environmental quality [12,13], stimulating physical activities [14,15,16], and encouraging social integration in communities [17,18]. 

Due to economic growth, improved health care services, and the decline in rural population, the incidences of epidemics, such as dysentery, tuberculous, and malaria, have been observed to be decreasing worldwide [19,20,21]. However, the heterogeneity in geography and socioeconomic conditions still leads to hot spots with high disease incidences [22,23,24,25,26]. The persistence and re-emergence of epidemics in recent decades have been associated with the process of global urbanization, which has been reshaping the environmental, economic, and demographic system [27]. Moreover, current climate change crises contemporarily affecting temperature, precipitation, flood, and extreme weather events are exacerbating the outbreak and spread of infectious diseases [28]. 

**Figure 1:**
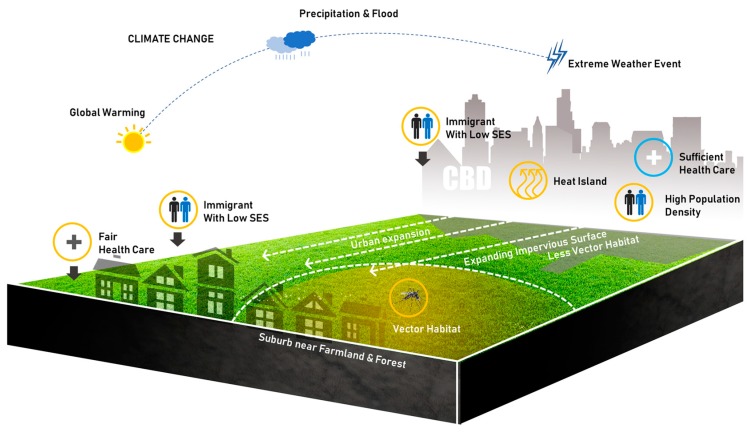
Urban Expansion, Epidemics, and Climate Change.

The interrelationship between climate change, urbanization, epidemics, and green space remains complex (Figure 1). Dysentery has been proved related to population density, high temperature, and sanitary conditions [29], while urbanization has also brought new challenges in controlling tuberculosis aroused by poverty and immigration [30,31,32,33], as well as human mobility [34,35]. Increasing impervious area during urban expansion may shrink the habitat of malaria vectors [36], while the immigrants or new residents with low socioeconomic status may face significant challenges of malaria for entering the territory of vectors in new cities located near farmlands, forests, and rivers [37,38].

Few studies have examined the correlation between green space and these three kinds of epidemics that have been proven relevant to vegetation in the urbanization background. 

Environment factors play an important role in dysentery infections, such as water pollution [39], distance to farmland [40], flood [41,42], temperature, and humidity [43,44,45]. In addition, people in urban environments are more vulnerable to temperature increases than rural populations [46]. Tuberculosis is caused by airborne particles from person to person and is affected by population density, which may share the same relevance of green space with dysentery [47]. Such factors pose a great challenge for public facilities with high population density [48,49], which may include public parks. Beyond that, the other two environmental factors, polluted water and cold weather, are also associated with tuberculosis [50,51]. 

The role of green space in malaria remains blurred compared to the possible benefits of dysentery and tuberculosis. Malaria is caused by infective mosquitoes which habitat mainly located in green spaces [52], while geographical environments provides the habitat for both vectors and humans, affecting the incidence and seasonality of malaria with different probabilities of infection and transmission [53]. Urban green space may be reserved as a livable environment for mosquitoes, such as ponds and shrubs [54,55]. Some studies report that the vegetation is not significantly associated with malaria [56,57], while others verify the positive effect of green space on mitigating temperature, which plays a critical role in malaria outbreak [58,59,60,61,62].

**Figure 2:**
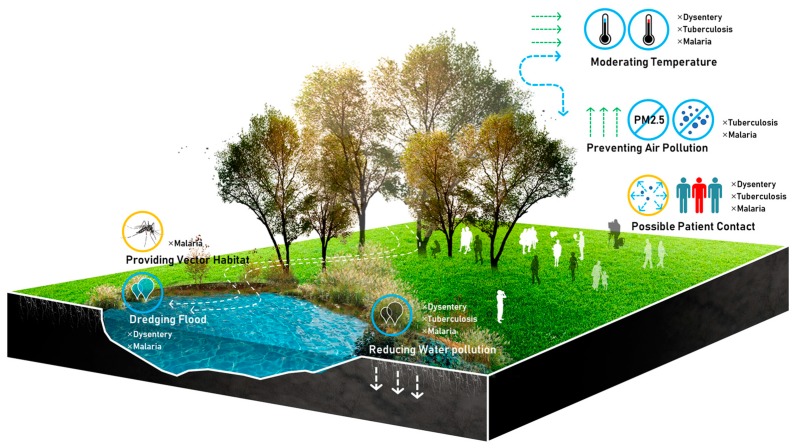
Dysentery, Tuberculosis, Malaria, and Green Space.

All the environmental factors influencing these three kinds of epidemics may be directly or indirectly affected by green space, as more and more studies have shown that green space can alleviate air and water pollution [63,64,65], moderate heat island effect [66], and control flood and waterlogging [67]. Much uncertainty still exists about the relationship between green space and epidemics since green space has been an important policy in building a healthy city (Figure 2).

Furthermore, it is necessary to understand the different mechanisms that various kinds of green spaces play in public health, as green space has been considered a combination of urban green space, agricultural space, and natural green space [68]. Many studies insist that the distance to the general green space plays a crucial role in public health [69]. More research has shown that it is parks that impose a more positive effect on the health condition of nearby residents [70,71,72,73], for it can largely increase physical activities [74,75]. Neighborhood green space, as a component of green space coverage, has also been verified to have positive effects on the surrounding population [76]. Different kinds of green spaces vary not only in terms of scale, function, and accessibility but also in terms of vegetation cover, ecological dimension, and environmental quality [77]. In the annual official reports of China Statistical Yearbook (http://www.stats.gov.cn), the Chinese government has been using three similar statistic indicators: Area of Green Space, Area of Parks, and Green Coverage ratio of built-up area. However, seldom have the different influences on public health imposed by these three kinds of green space been investigated and compared, and comparison would help to understand the role of green space in epidemic diseases and public health. 

This paper aims to provide a spatiotemporal overview of green space and epidemic diseases in China, in order to explore the correlation between them and to compare the different effects of these three kinds of green space on the macro scale with provincial statistical data of China in the past decade. The quantitative method adopted is the Panel Data Model (PDM), which provides three models to examine the longitudinal data, superior to traditional statistical analysis [78].

## 2. Materials and Methods

### 2.1. Variables and Data Source

**Figure 3:**
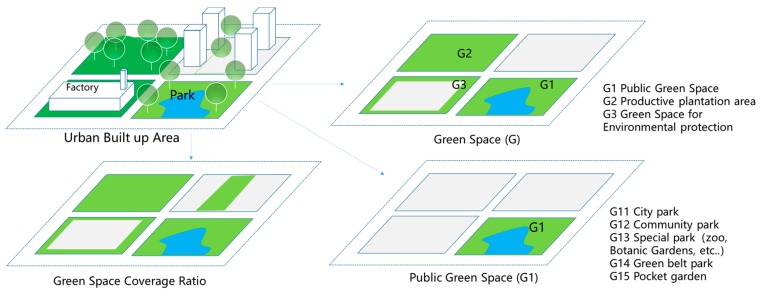
Three kinds of Green Space in Statistics Yearbook of China.

(1) Epidemic disease incidence. Three typical diseases were chosen as the dependent variables: Dysentery, Tuberculosis, and Malaria. The data was extracted from China Health Statistics Yearbook, 2007–2016 (http://www.nhc.gov.cn/). The unit of incidence is based on 1/100,000 people. A natural log-transformation was applied to the dependent variables to avoid the heteroscedasticity.

(2) Green space indicators and socioeconomic variables. All data are available in China Statistic Yearbook, 2007–2016 (http://www.stats.gov.cn/). The three statistic indicators, Area of Green Space, Area of Parks, and Green Coverage ratio of built-up area, are the corresponding variables of Green Space (GS), Public Green Space (Public GS) and Green Space Coverage Ratio (GS coverage). Figure 3 illustrates the three definitions of green space. 

Considering that economic factors and medical services may also affect the public health [79,80], several socioeconomic indicators were also selected as ancillary variables, such as total population (Pop), urban population (Urban Pop), population density (Pop density), built-up area (Built-up), gross domestic product (GDP), and medical workers (Medi_workers). Similarly, the natural log-transformation was applied to all the above variables.

(3) Temperature and Humidity. Since temperature and humidity could affect the incidence of epidemics, the PDM also added variables of temperature, such as annual average temperature (Average T), maximum temperature (High T), lowest temperature (Low T), and relative humidity (Humidity). The data is available from the National Meteorological Information Center (http://data.cma.cn/).

The panel data consists of indicators of 31 provinces, autonomous regions, and municipalities in ten-year, so we have 310 observations (31 × 10 = 310).

### 2.2. Methods

(1) Moran’s *I*

To explore the spatial distribution of green space and epidemic diseases, this article used Moran’s *I* as a measure of spatial autocorrelation. Moran’s *I* is defined as:

(1) $$I=\frac{N}{W}\frac{\sum_{i}\sum_{j}w_{ij}\left(x_{i}-\bar{x}\right)\left(x_{j}-\bar{x}\right)}{\sum_{i}{\left(x_{i}-\bar{x}\right)}^{2}}$$

where *N* is the number of spatial units indexed by *i* and *j*, *x* is an observed variable, $\bar{x}$ is the mean of *x*, *w_ij_* is a matrix of spatial weights, and *W* is the sum of all *w_ij_*. The value of Moran’s *I* is between −1 to 1, showing whether the spatial distribution of such variable is dispersed, clustered, or random.

(2) PDM

PDM is a popular quantitative method for longitudinal data in social science, epidemiology, and econometrics, which increases the estimator precision by increasing the number of observations and obtain more dynamic information than a single cross-sectional data [81]. PDM contains three kinds of models, the Pooled Effects model (PE), Fixed Effects model (FE), and random effects model (RE), wherein the FE model can eliminate the influence of individual-variant but time-invariant unobserved confounders. A common panel data regression model can be described through suitable restrictions of the following general model:

*y_it_* = *α_it_* + *X_it_β_it_* + *ε_it_* (2)

where *y* is the explained variable, *X* is the explanatory variable, *α* is the intercept, *β* is coefficients, *i* and *t* are indices for individuals and time, and *ε* is the error.

In the PE model, for all *i* and *t*, α*_it_* = α and *β*_it_ = *β*, that is:

*y_it_* = *α* + *X_it_β* + *ε_it_* (3)

In the FE model, for all *t*, α*_it_* = α, that is:

*y_it_* = *α_i_* + *X_it_β* + *ε_it_* (4)

In the RE model, *ε_it_* is assumed to vary stochastically over *i* and *t* requiring special treatment of the error variance matrix. To determine which model should be chosen, all the models need to be applied respectively. The Breusch–Pagan Lagrange Multiplier test should be implemented on the result of the PE model to decide whether the PE is the fittest, and the F-test can also be used to choose between FE and PE. The selection for fixed and random effects specifications is based on the Hausman-type test [82].

## 3. Results

### 3.1. General Description of Green Space and Diseases

In general, decreasing trends over time were observed in all the three epidemic diseases with the average incidence rate of Dysentery, Tuberculosis, and Malaria decreasing from 40.51 to 12.99, 91.84 to 68.85, and 3.81 to 0.19, respectively (Table 1). In contrast, it was found that all kinds of green spaces expanded, and the mean logarithm value of Green Space and Public Green Space correspondingly increased from 5.56 to 8.98 and 1.08 to 2.10, and the mean green space coverage ratio increased from 34.18% to 39.17%.

**Figure 4:**
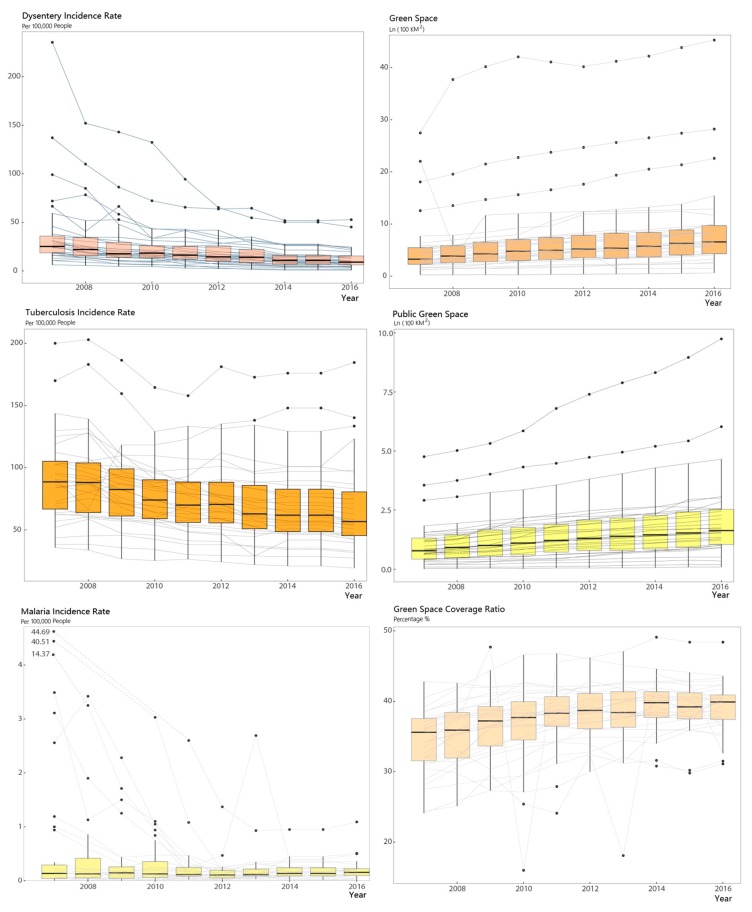
Descriptive statistics of variables.

Figure 4 displays the results of descriptive statistics for the variables used in this paper. On the left side are the variables of Dysentery, Tuberculosis, and Malaria, with indicators of green spaces are on the right side. The lines therein illustrate the time-serial data of the 31 provinces in China from 2007 to 2016; the boxplots give the quartiles of corresponding descriptive statistics. 

### 3.2. Spatial Distribution of Green Space and Diseases

**Figure 5:**
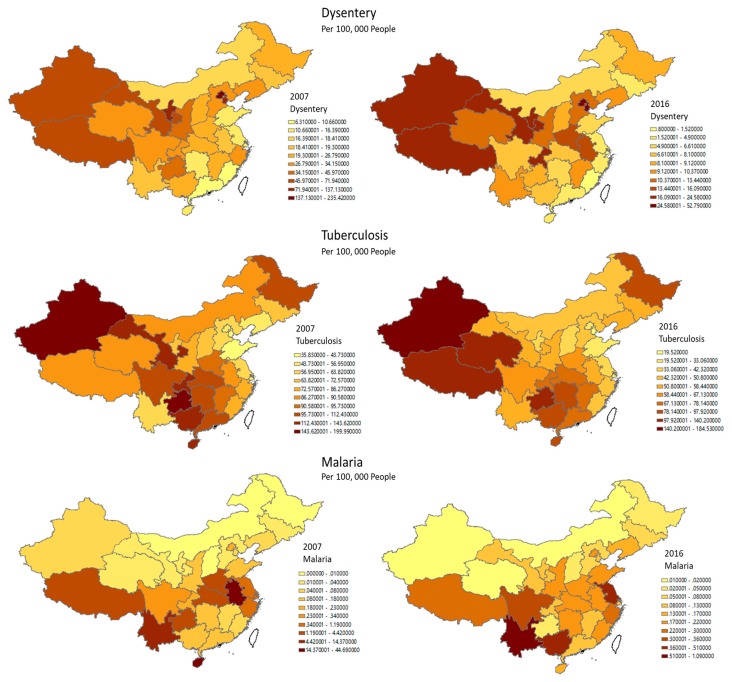
Geographical distribution of epidemic diseases.

Figure 5 illustrated the spatial distribution of the incidence rates of three diseases in 2007 and 2016, which indicates that the incidence rates of Dysentery and Tuberculosis show obvious spatial clustered patterns. Such interpretations can also be verified in the spatial autocorrelation via Global Moran’s *I* statistics in Table 2. There existed a clear trend of increasing Moran’s *I* in Dysentery incidence, but a slight decreasing in Tuberculosis incidence from 2007 to 2016 in China; both results were significant at the *p* = 0.001 level. However, no obvious spatial autocorrelation was detected in the incidence of Malaria.

**Figure 6:**
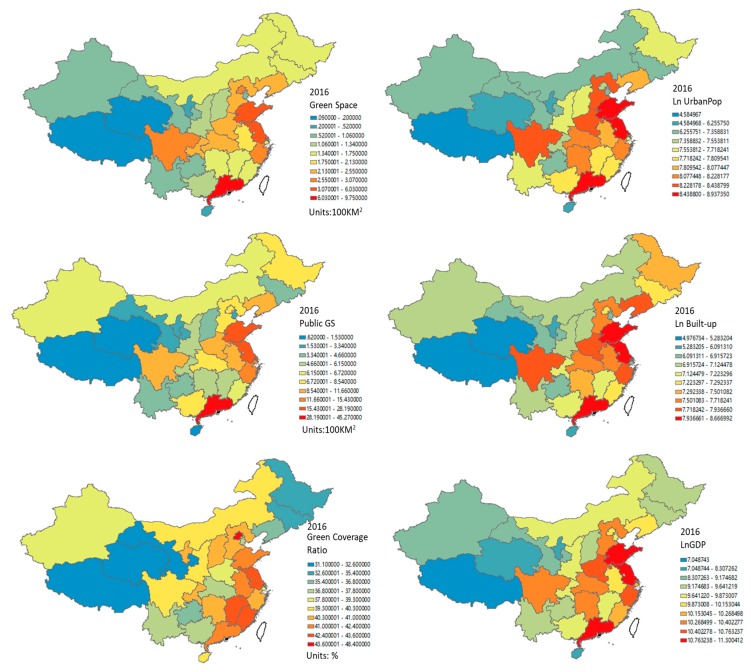
Geographical distribution of green space and other control variables in 2016.

Further, Global Moran’s *I* statistics of control variables in Table 3 reveals that only the green space coverage ratio displayed a cluster pattern in the three kinds of green space statistics. Spatial autocorrelation was also observed in the logarithm value of Urban Population, Built-up area, and GDP, which share a similar hierarchical distribution in Figure 6.

### 3.3. Correlation Test and Panel Data Analysis

**Figure 7:**
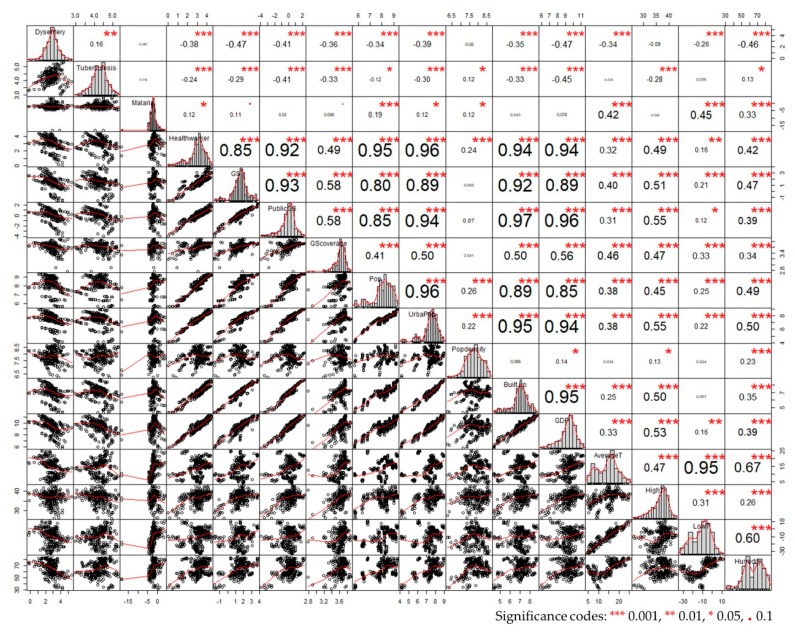
Correlation analysis of all variables by R. Note: The distribution of each variable is shown on the diagonal, the bivariate scatter plots with a fitted line are on the left side of the diagonal, and the values of the correlation plus the significance level as stars are on the right side.

The results in Figure 7 provide an overview correlation of all variables by R with package Performance Analytics. The chart is quite revealing in several ways. Firstly, all variables, except Malaria and Population density, presented a noteworthy correlation with others. Furthermore, most of the control variables contributed to a negative correlation with the incidences of the three diseases. The temporal variables were significantly associated with other explanatory variables, imposing different effects on diseases and sharing a similar situation with indicators of green spaces. However, the approximately straight lines in scatter plots implied there existed multi-collinearity among variables, which can be straightforwardly observed in variables of Urban Pop, Built-up, and GDP. A further stepwise regression combining with the PDM was taken to explore the influence of multicollinearity. 

The General regression model with a logarithm transformation of epidemic incidence could be written as:

*ln(incidence) = a + β*_1_*ln(Healthworker) + β_2_*ln(GS) + β_3_*ln(PublicGS) + β_4_*ln(GScoverage) + β_5_*ln(Pop) + β_6_*ln(UrbaPop) + β_7_*ln(Popdensity) + β_8_*ln(Built.up) + β_9_*ln(GDP) + β_10_*AverageT + β_11_*HighT + β_12_*LowT + β_13_*Humidity + ε (5)

Based on the PDM, the three models of Pooled Effects (PE), Fixed Effects (FE), and Random Effects (RE) should been applied and compared by Lagrange Multiplier Test, *F* Test, and Hausman Test. The result in Table 4 indicated that the FE model was better for Dysentery, while the RE models were better for Tuberculosis and Malaria.

All the following tables (Table 5, Table 6 and Table 7) provide results of traditional PDM and PDM with stepwise regression, indicating that the stepwise regression can also be applied in PDM to eliminate the multicollinearity and optimize the regression model. 

In Table 5, the results of the FE model of Dysentery reveal that only the indicator of general green space shows the connection with dysentery. Among socioeconomic variables, Population, GDP, and Medical workers are negatively correlated with the incidence of dysentery, while Urban population and Built-up are positive. Average temperature and Humidity also play an important role in reducing the incidence of dysentery. A further step of stepwise regression to eliminate multicollinearity retains only three variables with decreased Variance Inflation Factors (VIF): the number of medical workers, general green space, and relative humidity. For every 1% increase in the number of medical workers and green space area, the incidence of dysentery will decrease by 1.225% and 0.235%, respectively, and for every 1% increase in relative humidity, the incidence will decrease by 0.023%.

In Table 6, the results of the RE model of Tuberculosis show that all the three green space indicators impose significant effects on tuberculosis, wherein GS and Public GS are negative, and GS coverage is positive. An increase of Population, GDP, Urban population, and Lowest temperature may enlarge the risk of tuberculosis, while Medical workers, Built-up area, average temperature, and relative humidity would reduce the risk. Further stepwise regression keeps five variables, in which GS, Population, and Humidity are positive, and GDP and average temperature are negative. After the process of multicollinearity elimination, GS, GDP, and Humidity show opposite correlations. For every 1% increase in population and relative humidity, the incidence of Tuberculosis would increase by 0.425% and 0.011%, while for GDP, Tuberculosis would decrease by 0.619%. An average temperature increase of one Celsius degree will result in a reduction of Dysentery by 0.014%.

The results of the RE model of Malaria in Table 7 indicate that only Public GS and Highest temperature are associated with the incidence of malaria. In the stepwise regression operation, four variables remained, wherein GS, Pop Density, and Low T are positive, and yet High T is negative.

## 4. Discussion

The initial purpose of this research was to compare the correlations between various kinds of green spaces and epidemics. An unexpected finding is that all three kinds of green space display different correlations with epidemics in PDM, while the results in PDMSR further indicate that only GS is significantly correlated with the three epidemics. This result can be explained by the apparent collinearity between variables of Medical workers, Population, Urban Population, Built-up Area, and GDP in Figure 7, ranging from 0.85 to 0.96.

Table 8 also shows that green space has different effects on dysentery, tuberculosis, and malaria in China, which has not been previously described. An increase in green space would help reduce the incidence of dysentery, while increasing the risk of Tuberculosis and Malaria.

Such a difference may be explained by increasing green space along the process of urban expansion in China. Urban expansion with a better socioeconomic condition will largely reduce the incidence of dysentery by providing better sanitary services [29]. The negative correlation of GDP in PDM of Tuberculosis also demonstrates the importance of the economy, whereas the positive correlation of Green space and Population suggest that population increases in urbanization process may raise the risk of tuberculosis [48,49]. A similar demographic influence can be observed in the PDM of Malaria, wherein variables of green space and population density are significantly positively correlated, as opposed to previous findings that there existed no correlation between green space and malaria [56,57]. The result could also be related to the potential habitat for mosquitoes provided by green space [55].

Urban expansion on the edge of the city will lead to a simultaneous increase in green space and population, and immigrants with low socioeconomic status may face the threat of epidemics and inadequate medical services. Such results are consistent with previous studies on urbanization and epidemiology [27]. Moreover, socioeconomic development still plays a crucial role in controlling epidemics, which is in line with expectations in the previous literature that urbanization would lead to higher economic conditions, better health care services, and more green space, benefiting the control of epidemics [19,20,21]. The geographical distribution of green space and other socioeconomic variables in Figure 6 and the Global Moran’s *I* index in Table 2 also provide a supplementary explanation for this result. Most of the Chinese provinces with high GDP are located along the coast of China, which have a better quality of green space and other health facilities. 

What is surprising is that the models of Tuberculosis and Malaria exclude the variable of medical workers representing the level of health care service, and this result seems to be contrary to most literature [30]. A possible explanation may be that the incidence of these two epidemics has less connection with medical care conditions, more with the heterogeneous environment of various contagious probability [28]. 

In terms of temperature and humidity variables, only relative humidity has a negative impact on dysentery, contrary to previous literature which argues that high temperature and humidity can lead to larger dysentery incidence [43]. Such results may be related to the provincial average relative humidity value used in this study. As for tuberculosis, the result confirms that cold weather may increase the likelihood of pathogenic exposure, while higher humidity may contribute to the spread of tuberculosis [51]. In addition, it has been confirmed that temperature parameters are associated with malaria as the minimum and maximum temperature are highly correlated with Malaria. Since the minimum temperature is significantly negatively correlated with Normalized Difference Vegetation Index( NDVI) [61], the impact of green space on malaria may be further enhanced. 

## 5. Conclusions

The main contribution of this paper is that we explored the different relations between epidemics and the three kinds of green space, which had rarely been explored and compared together. The result provides more meaningful guidance for public policy on the construction of green space correspondingly. Furthermore, a PDM with stepwise regression can alleviate the influence of multicollinearity and provide more accurate correlations between the variables. 

As the indicators of green space become more and more popular in evaluating the construction of healthy cities, this article argues that the increase of green space will not straightforwardly lead to a better healthy condition, especially in the process of urban expansion. The government should pay more attention to the quality of green space to improve physical activities of residents, ensure enough sanitary facilities, and reduce mosquito habitat in green space by appropriate landscape design and technologies. 

However, the study does have limitations. First, the main weakness of the current study is taking the provincial boundary as geographical units, which limits the resolution of the sample size. Second, although spatial autocorrelation had been pointed out, spatial panel model could be applied to taking spatial weight into account. Another exploration worth discussing may be the link between green space and the infection channels of diseases. For instance, dysentery is transmitted through human contact, food, polluted water, and flies, while tuberculosis and malaria are mainly infected by respiratory tract and mosquitos, respectively. Therefore, more detailed studies would be necessary to explore how green space influence the infection channels of diseases.

## Figures and Tables

**Table 1:** Descriptive statistics of variables. GDP = gross domestic product.

**1–1 Epidemics Indices**										
**Dysentery** (Incidence per 100,000 person)										
**Year**	**2007**	**2008**	**2009**	**2010**	**2011**	**2012**	**2013**	**2014**	**2015**	**2016**
Min.	6.31	5.66	4.64	4.49	2.84	2.45	1.62	0.86	0.86	0.80
Median	25.20	22.08	17.75	18.31	16.31	14.51	14.03	10.87	10.87	9.12
Mean	40.52	32.93	28.60	24.67	22.61	19.52	17.61	14.44	14.44	12.99
Max.	235.42	152.01	143.01	132.37	94.45	65.32	64.52	51.79	51.79	52.79
S.E.	45.52	32.09	28.42	24.21	18.90	15.40	14.31	12.29	12.29	11.65
**Tuberculosis** (Incidence per 100,000 person)										
**Year**	**2007**	**2008**	**2009**	**2010**	**2011**	**2012**	**2013**	**2014**	**2015**	**2016**
Min.	35.83	33.98	26.91	25.54	26.69	24.41	22.28	21.27	21.27	19.52
Median	88.64	88.11	82.48	74.07	69.91	70.52	62.90	61.84	61.84	56.66
Mean	91.84	91.39	84.41	77.80	75.23	75.64	71.11	70.42	70.42	68.85
Max.	199.99	202.93	186.35	164.46	157.83	181.17	172.73	176.00	176.00	184.53
S.E.	36.46	37.84	33.09	28.64	29.24	33.05	33.50	34.14	34.14	36.47
**Malaria** (Incidence per 100,000 person)										
**Year**	**2007**	**2008**	**2009**	**2010**	**2011**	**2012**	**2013**	**2014**	**2015**	**2016**
Min.	0.00	0.01	0.00	0.01	0.02	0.01	0.03	0.01	0.01	0.01
Median	0.16	0.13	0.16	0.12	0.11	0.10	0.11	0.13	0.13	0.15
Mean	3.82	2.11	1.08	0.49	0.26	0.16	0.24	0.18	0.18	0.20
Max.	44.69	22.04	9.65	4.63	2.60	1.37	2.69	0.95	0.95	1.09
S.E.	10.72	5.53	2.38	0.97	0.48	0.25	0.48	0.18	0.18	0.21
**1–2 Three Kinds of Green Space Indices**										
**Green Space** (Units: 100 km^2^)										
**Year**	**2007**	**2008**	**2009**	**2010**	**2011**	**2012**	**2013**	**2014**	**2015**	**2016**
Min.	0.20	0.20	0.22	0.21	0.29	0.34	0.36	0.42	0.53	0.62
Median	3.26	3.85	4.29	4.79	4.98	5.18	5.36	5.74	6.31	6.56
Mean	5.56	5.69	6.43	6.89	7.24	7.64	7.83	8.15	8.61	8.99
Max.	27.47	37.70	40.16	42.04	41.06	40.17	41.20	42.19	43.84	45.27
S.E.	6.26	7.05	7.60	7.94	7.87	7.86	8.19	8.39	8.64	8.93
**Public Green Space** (Units: 100 km^2^)										
**Year**	**2007**	**2008**	**2009**	**2010**	**2011**	**2012**	**2013**	**2014**	**2015**	**2016**
Min.	0.02	0.03	0.04	0.03	0.05	0.05	0.06	0.07	0.09	0.09
Median	0.78	0.91	1.00	1.10	1.21	1.30	1.39	1.45	1.53	1.63
Mean	1.09	1.18	1.30	1.42	1.56	1.67	1.77	1.86	1.98	2.11
Max.	4.76	5.02	5.32	5.85	6.80	7.40	7.89	8.32	8.96	9.75
S.E.	1.03	1.07	1.14	1.23	1.36	1.46	1.55	1.63	1.74	1.88
**Green Space Coverage Ratio** (Units: %)										
**Year**	**2007**	**2008**	**2009**	**2010**	**2011**	**2012**	**2013**	**2014**	**2015**	**2016**
Min.	24.10	25.10	27.30	16.00	24.10	30.00	18.10	30.80	29.80	31.10
Median	35.60	35.90	37.20	37.70	38.30	38.70	38.40	39.80	39.20	39.90
Mean	34.18	35.32	36.70	36.33	37.75	38.46	38.13	39.34	39.11	39.17
Max.	42.80	42.60	47.70	46.60	46.80	46.20	47.10	49.10	48.40	48.40
S.E.	4.51	4.54	4.80	6.03	4.66	3.83	5.24	3.76	3.68	3.67
**1–3 Socioeconomic Variables**										
**Medical Workers** (Units: 10,000 People)										
**Year**	**2007**	**2008**	**2009**	**2010**	**2011**	**2012**	**2013**	**2014**	**2015**	**2016**
Min.	1.02	1.17	1.60	1.67	2.22	2.16	2.47	2.65	2.91	2.92
Median	17.51	18.35	22.35	23.09	24.46	25.96	26.98	28.28	29.16	30.17
Mean	19.06	19.90	25.10	26.44	27.76	29.38	31.55	32.98	34.46	36.01
Max.	45.21	47.98	60.21	64.59	68.96	73.89	81.93	83.85	85.57	87.41
S.E.	11.49	12.02	15.62	16.55	17.47	18.57	20.24	20.99	21.78	22.67
**Population** (Units: 10,000 People)										
**Year**	**2007**	**2008**	**2009**	**2010**	**2011**	**2012**	**2013**	**2014**	**2015**	**2016**
Min.	289.0	292.0	296.0	300.0	303.0	308.0	312.0	318.0	324.0	331.0
Median	3708.0	3718.0	3727.0	3735.0	3743.0	3753.0	3774.0	3806.0	3812.0	3813.0
Mean	4206.0	4240.0	4272.0	4303.0	4324.0	4348.0	4371.0	4395.0	4422.0	4451.0
Max.	9660.0	9893.0	10,130.0	10,441.0	10,505.0	10,594.0	10,644.0	10,724.0	10,849.0	10,999.0
S.E.	2706.2	2725.7	2748.3	2765.0	2769.4	2778.8	2785.7	2797.8	2817.2	2843.1
**Urban Population** (Units: 10,000 People)										
**Year**	**2007**	**2008**	**2009**	**2010**	**2011**	**2012**	**2013**	**2014**	**2015**	**2016**
Min.	62.0	64.0	66.0	68.0	69.0	70.0	74.0	82.0	90.0	98.0
Median	1728.0	1820.0	1904.0	1844.0	1942.0	2038.0	2115.0	2173.0	2116.0	2148.0
Mean	1936.0	2005.0	2068.0	2166.0	2236.0	2311.0	2371.0	2432.0	2500.0	2574.0
Max.	6099.0	6269.0	6423.0	6910.0	6986.0	7140.0	7212.0	7292.0	7454.0	7611.0
S.E.	1314.5	1352.7	1389.5	1479.3	1511.2	1550.6	1579.2	1608.2	1656.3	1707.7
**Population Density** (Units: person per km^2^)										
**Year**	**2007**	**2008**	**2009**	**2010**	**2011**	**2012**	**2013**	**2014**	**2015**	**2016**
Min.	622.0	649.0	902.0	575.0	515.0	1032.0	1059.0	1291.0	1336.0	1145.0
Median	2376.0	2370.0	2344.0	2428.0	2487.0	2674.0	2570.0	2604.0	2557.0	2624.0
Mean	2607.0	2642.0	2634.0	2707.0	2725.0	2805.0	2818.0	2850.0	2776.0	2809.0
Max.	5938.0	5967.0	5530.0	5506.0	5821.0	5483.0	5541.0	5474.0	5504.0	5244.0
S.E.	1341.8	1353.3	1248.7	1343.8	1322.8	1238.1	1193.0	1184.3	1121.3	1084.2
**Built-Up Area** (Units: km^2^)										
**Year**	**2007**	**2008**	**2009**	**2010**	**2011**	**2012**	**2013**	**2014**	**2015**	**2016**
Min.	79.0	79.0	81.0	85.0	90.0	120.0	120.0	126.0	145.0	145.0
Median	886.0	885.0	950.0	1038.0	1077.0	1133.0	1206.0	1231.0	1329.0	1371.0
Mean	1144.2	1199.8	1259.9	1334.1	1406.6	1470.0	1543.6	1605.5	1681.0	1753.0
Max.	4084.0	4133.0	4434.0	4618.0	4829.0	5026.0	5232.0	5398.0	5633.0	5808.0
S.E.	882.5	882.5	962.3	1011.8	1058.9	1100.8	1147.1	1193.4	1238.9	1289.6
**GDP** (Units: 100 Million RMB)										
**Year**	**2007**	**2008**	**2009**	**2010**	**2011**	**2012**	**2013**	**2014**	**2015**	**2016**
Min.	341.4	394.9	441.4	507.5	605.8	701.0	815.7	920.8	1026.0	1151.0
Median	6423.2	8314.4	8587.0	10,368.6	12,582.0	14,454.0	16,205.5	17,689.9	17,832.0	18,499.0
Mean	9023.8	10,752.1	11,784.0	14,098.1	16,820.7	18,598.0	20,462.8	22,075.8	23,315.0	25,164.0
Max.	31,777.0	36,796.7	39,482.6	46,013.1	53,210.3	57,068.0	62,474.8	67,809.9	72,813.0	80,855.0
S.E.	7647.3	7647.3	9730.4	11,401.3	13,216.3	14,326.0	15,709.7	16,987.7	18,219.0	20,103.1
**1–4 Temperature Variables**										
**Average Temperature** (Units: Celsius degree)										
**Year**	**2007**	**2008**	**2009**	**2010**	**2011**	**2012**	**2013**	**2014**	**2015**	**2016**
Min.	6.10	5.70	5.00	4.50	5.20	4.60	4.30	5.10	5.60	5.00
Median	15.60	14.90	14.90	14.60	14.20	14.30	15.80	15.40	15.20	15.80
Mean	14.95	14.33	14.42	14.09	13.88	13.83	14.38	14.41	14.56	14.60
Max.	24.10	23.40	24.30	24.60	23.30	24.60	24.30	24.70	25.30	24.60
S.E.	4.93	4.93	5.24	5.27	4.99	5.25	5.27	5.10	5.05	5.17
**Highest Temperature** (Units: Celsius degree)										
**Year**	**2007**	**2008**	**2009**	**2010**	**2011**	**2012**	**2013**	**2014**	**2015**	**2016**
Min.	29.00	26.60	30.40	29.60	27.50	28.90	28.10	29.30	29.00	26.20
Median	37.40	37.10	37.50	38.40	36.80	36.90	37.10	37.00	37.30	37.00
Mean	36.54	36.02	36.94	37.76	36.31	35.99	36.82	36.76	36.77	36.53
Max.	39.80	39.70	42.10	41.80	42.70	41.00	41.60	42.80	40.60	42.30
S.E.	3.01	3.01	3.13	2.78	3.05	3.15	3.32	2.96	2.72	3.18
**Lowest Temperature** (Units: Celsius degree)										
**Year**	**2007**	**2008**	**2009**	**2010**	**2011**	**2012**	**2013**	**2014**	**2015**	**2016**
Min.	−24.00	−28.00	−32.30	−31.80	−32.50	−31.30	−33.30	−32.70	−29.60	−31.40
Median	−5.00	−10.20	−8.60	−8.10	−8.60	−7.60	−8.30	−6.40	−6.20	−9.80
Mean	−7.13	−10.62	−10.41	−10.64	−10.83	−10.70	−10.90	−9.81	−8.34	−12.30
Max.	10.70	8.00	9.00	8.40	7.70	9.20	8.90	8.30	11.00	5.60
S.E.	9.29	9.29	10.72	11.00	10.70	10.82	10.68	9.86	9.71	9.62
**Relative Humidity** (Units: %)										
**Year**	**2007**	**2008**	**2009**	**2010**	**2011**	**2012**	**2013**	**2014**	**2015**	**2016**
Min.	29.00	38.00	31.00	33.00	34.00	34.00	38.00	36.00	34.00	37.00
Median	37.40	64.00	66.00	69.00	67.00	67.00	68.00	63.00	64.00	69.00
Mean	36.54	63.65	63.58	64.68	63.35	65.10	64.58	65.32	66.52	67.06
Max.	39.80	82.00	81.00	81.00	81.00	85.00	82.00	83.00	84.00	83.00
S.E.	3.01	10.79	11.19	11.73	11.05	12.69	11.27	12.29	12.49	12.13

**Table 2:** Global Moran’s *I* of epidemic diseases incidences from 2007 to 2016.

Year	Dysentery			Tuberculosis			Malaria		
	Moran’s *I*	*p*	*z*	Moran’s *I*	*p*	*z*	Moran’s *I*	*p*	*z*
2007	0.196	<0.001	3.514	0.380	<0.001	5.480	−0.050	0.789	−0.266
2010	0.192	<0.001	3.578	0.391	<0.001	5.632	0.004	0.561	0.580
2013	0.221	<0.001	3.468	0.367	<0.001	5.326	0.023	0.253	1.141
2016	0.232	<0.001	3.680	0.338	<0.001	4.978	0.023	0.381	0.875

**Table 3:** Global Moran’s *I* of control variables in 2016.

Variables	GS	Public GS	GS_Coverage	Medical Worker	Pop	Pop_Density	Urban_Pop	Built-up	GDP
Moran’s *I*	0.015	−0.023	0.165	0.014	0.092	0.087	0.154	0.113	0.216
*p*	0.469	0.888	0.009	0.538	0.098	0.485	0.012	0.052	0.001
*z*	0.722	0.139	2.605	0.615	1.651	−0.696	2.518	1.942	3.289

**Table 4:** *p*-values of Lagrange Multiplier Test, *F* Test, and Hausman Test.

PDM	Lagrange Multiplier Test	*F* Test	Hausman Test
Dysentery	<0.001	<0.001	<0.001
Tuberculosis	<0.001	<0.001	0.1047
Malaria	<0.001	<0.001	0.172

**Table 5:** Results of Panel Data Model (PDM) of Dysentery.

Variable	PDM with Stepwise Regression							
	*β*	S.E.	*p*	VIF	*β*	S.E.	*p*	VIF
Medi_Workers	−0.632	0.206	0.002 **	38.23	−1.225	0.100	0.000 ***	3.67
GS	−0.158	0.074	0.033 *	11.23	−0.235	0.078	0.002 **	3.88
Public GS	0.282	0.190	0.140	48.79				
GS Coverage	0.059	0.176	0.737	2.18				
Pop	−3.207	0.623	0.000 ***	62.47				
Urban Pop	1.716	0.561	0.002 **	99.38				
Pop Density	−0.026	0.089	0.769	1.54				
Built-up	0.740	0.316	0.020 *	47.23				
GDP	−1.244	0.158	0.000 ***	32.29				
Average T	−0.103	0.032	0.001 **	23.83				
High T	0.004	0.012	0.706	2.42				
Low T	0.007	0.007	0.366	19.49				
Humidity	−0.027	0.005	0.000 ***	2.94	−0.023	0.006	0.000 ***	1.29
Adjusted R^2^	0.591							
F statistics	159.764							
Overall significance of model	<0.001							

Significance codes: *** 0.001, ** 0.01, * 0.05. VIF: Variance Inflation Factors.

**Table 6:** Results of PDM of Tuberculosis.

Variable	PDM with Stepwise Regression							
	*β*	S.E.	*p*	VIF	*β*	S.E.	*p*	VIF
(Intercept)	−0.175	0.080	0.030		5.897	0.324	0.000 ***	
Medi_Workers	−1.109	0.137	0.000 ***	38.23				
GS	−3.951	0.556	0.000	11.23	0.127	0.040	0.002 **	5.27
Public GS	−0.025	0.006	0.000 ***	48.79				
GS Coverage	7.072	1.180	0.000 ***	2.18				
Pop	0.489	0.110	0.000 ***	62.47	0.425	0.042	0.000 ***	4.15
Urban Pop	0.181	0.051	0.000 ***	99.38				
Pop Density	−0.061	0.105	0.559 .	1.54				
Built−up	−0.277	0.159	0.083	47.23				
GDP	0.604	0.141	0.000 ***	32.29	−0.619	0.044	0.000 ***	6.68
Average T	−1.031	0.162	0.000 ***	23.83	−0.014	0.005	0.002 **	1.85
High T	0.034	0.040	0.391	2.42				
Low T	0.280	0.121	0.022 *	19.49				
Humidity	−0.497	0.083	0.000 ***	2.94	0.011	0.002	0.000 ***	2.10
Adjusted R^2^	0.511							
Overall significance of model	<0.001							

Significance codes: *** 0.001, ** 0.01, * 0.05, **.** 0.1.

**Table 7:** Results of PDM of Malaria.

Variable	PDM with Stepwise Regression							
	*β*	S.E.	*p*	VIF	*β*	S.E.	*p*	VIF
(Intercept)	−14.057	7.566	0.064 .		0.364	2.023	0.857	
Medi_Workers	0.288	0.703	0.682	38.23				
GS	0.336	0.327	0.305	11.23	0.240	0.115	0.037 *	1.36
Public GS	−1.767	0.673	0.009 **	48.79				
GS Coverage	0.481	1.020	0.637	2.18				
Pop	0.176	0.903	0.845	62.47				
Urban Pop	−0.018	1.039	0.987	99.38				
Pop Density	0.305	0.256	0.236	1.54	0.565	0.211	0.008 **	1.02
Built-up	1.063	0.777	0.172	47.23				
GDP	0.287	0.530	0.589	32.29				
Average T	0.064	0.094	0.493	23.83				
High T	−0.145	0.050	0.004 **	2.42	−0.169	0.039	0.000 ***	1.45
Low T	0.062	0.042	0.141	19.49	0.098	0.010	0.000 ***	1.11
Humidity	0.004	0.014	0.788	2.94				
Adjusted R^2^	0.252							
Overall significance of model	<0.001							

Significance codes: *** 0.001, ** 0.01, * 0.05, **.** 0.1.

**Table 8:** Coefficients of Panne Data Model with Stepwise Regression (PDMSR).

Variables	Dysentery	Tuberculosis	Malaria
Medi_Workers	↓ −1.225 ***		
GS	↓ −0.235 **	↑ 0.127 **	↑ 0.240 *
Pop		↑ 0.425 ***	
Pop Density			↑ 0.565 **
GDP		↓ −0.619 ***	
Average T		↓ −0.014 **	
High T			↓ −0.169 ***
Low T			↑ 0.098 ***
Humidity	↓ −0.023***	↑ 0.011 ***	

correlation symbol: ↑ positive, ↓ negative; Significance codes: *** 0.001, ** 0.01, * 0.05, **.** 0.1.
